# Identifying key soil characteristics for *Francisella tularensis* classification with optimized Machine learning models

**DOI:** 10.1038/s41598-024-51502-z

**Published:** 2024-01-19

**Authors:** Fareed Ahmad, Kashif Javed, Ahsen Tahir, Muhammad Usman Ghani Khan, Mateen Abbas, Masood Rabbani, Muhammad Zubair Shabbir

**Affiliations:** 1 Department of Computer Science, University of Engineering and Technology, Lahore, Pakistan; 2 Quality Operations Laboratory, Institute of Microbiology, University of Veterinary and Animal Sciences, Lahore, Pakistan; 3 Institute of Microbiology, University of Veterinary and Animal Sciences, Lahore, Pakistan; 4 Department of Electrical Engineering, University of Engineering and Technology, Lahore, Pakistan

**Keywords:** Computational biology and bioinformatics, Machine learning, Infectious-disease diagnostics, Infectious-disease epidemiology, Diagnosis, Disease prevention

## Abstract

*Francisella tularensis* (Ft) poses a significant threat to both animal and human populations, given its potential as a bioweapon. Current research on the classification of this pathogen and its relationship with soil physical–chemical characteristics often relies on traditional statistical methods. In this study, we leverage advanced machine learning models to enhance the prediction of epidemiological models for soil-based microbes. Our model employs a two-stage feature ranking process to identify crucial soil attributes and hyperparameter optimization for accurate pathogen classification using a unique soil attribute dataset. Optimization involves various classification algorithms, including Support Vector Machines (SVM), Ensemble Models (EM), and Neural Networks (NN), utilizing Bayesian and Random search techniques. Results indicate the significance of soil features such as clay, nitrogen, soluble salts, silt, organic matter, and zinc , while identifying the least significant ones as potassium, calcium, copper, sodium, iron, and phosphorus. Bayesian optimization yields the best results, achieving an accuracy of 86.5% for SVM, 81.8% for EM, and 83.8% for NN. Notably, SVM emerges as the top-performing classifier, with an accuracy of 86.5% for both Bayesian and Random Search optimizations. The insights gained from employing machine learning techniques enhance our understanding of the environmental factors influencing Ft’s persistence in soil. This, in turn, reduces the risk of false classifications, contributing to better pandemic control and mitigating socio-economic impacts on communities.

## Introduction

Bacteria live within us, on us, and in the environment. While many bacteria coexist harmlessly, certain pathogenic strains pose indirect threats to human health through their impact on plants, birds, and animals, as revealed by several zoonotic infections^1^. These infections can quickly spread from animals to humans with or without a mechanical or biological vector^2^. Zoonoses represent a significant global challenge, contributing to 61% of prevailing and 75% of emerging human infections, along with billions of dollars of economic loss in developed countries like the US and Canada^3^

Among these zoonotic threats is tularemia, induced by the highly contagious intracellular bacterium *Francisella tularensis* (Ft). While mostly prevalent in the northern hemisphere, only ten organisms of the Ft can cause the disease^4^. Classified as a Category A biological agent by the Centers for Disease Control and Prevention (CDC), Ft has the potential for biowarfare due to its ease of propagation and high morbidity and mortality rates^5^. The bacterium exists in four subspecies, with Ft (Type-A) recognized as particularly hazardous, resulting in mortality rates ranging from 30 to 60% among affected individuals. An expert committee convened by the World Health Organization (WHO) issued a stark prediction, highlighting the potential devastation of releasing aerosols containing 50 kg of Ft over a densely populated metropolis of 5 million inhabitants. The projected outcomes were alarming, anticipating 19,000 deaths and 250,000 illnesses as a consequence of such an aerosol exposure^6^.

Tularemia is prevalent throughout North America, Europe, Asia, and Australia. In Europe, the illness is widely prevailing. In 2019, almost 1500 cases of tularemia were reported in the European Union, with 56% of cases in Sweden, followed by Norway, which spread mainly via mosquito bites^7^. In Asian regions, including Japan, Turkey, Iran, China, Turkmenistan, Azerbaijan, Afghanistan, and Kazakhstan, instances of tularemia have been documented^8^. In the United States, the disease has established a nearly ubiquitous presence, with an average of 143 registered patients annually from 2005 to 2014. Subsequently, the numbers surged to 314, 230, 239, 229, 274, and 150 in the years 2015 through 2020^9^. Over the last two decades, outbreaks of tularemia have been reported not only in Asia but also in Japan, South Korea, the European Union, the United States, and Canada^10^.

The pathogen can persist for extended periods in various environments, including soil, moist hay, water, straw, and decaying animal carcasses^11^. The diverse modes of transmission, from contaminated food and water to inhalation of infected air, further complicate control measures^12^. This resilience poses a particular threat in regions lacking stringent biological waste handling standards, where infected materials can decompose and spread through natural elements.

Identifying Ft in soil presents a critical step in controlling disease outbreaks, but traditional identification techniques like Mass Spectrometry (MS)^13^, Polymerase Chain Reaction (PCR)^14^, and Enzyme-Linked Immunosorbent Assay (ELISA)^15^ are costly and time-consuming. This study builds on the understanding that soil attributes, such as moisture, pH, and mineral contents, can be crucial in screening samples positive for Ft, as studies^16–20^ indicate the pivotal role of these physicochemical factors in soil pathogen persistence. Moreover, recognizing the limited exploration of machine learning models in this domain, we embark on a novel approach to predict Ft prevalence in soil.

While existing research primarily relies on statistical methods, our study differentiates by employing machine learning models for predicting the epidemiological models for this soil-borne pathogen. Building upon our earlier work, which utilized neural networks for Ft classification and achieved a notable accuracy of 82.61%^21^, subsequent enhancements raised this accuracy to 84.35%, utilizing feature ranking and machine learning classifiers^22^. The present study aims to contribute to this evolving field by introducing a unique approach for feature ranking by assessing the rank of an attribute by utilizing the commulative score of all feature ranking methods to overcome any bias introducted by different ranking methods. Furthermore, to enhance the accuracy and efficiency of our model, we incorporate bayesian and random search optimization techniques, which help in finding the best hyperparameters for our machine learning model, ensuring optimal performance and robust prediction.

The outlined objectives include assessing classifier performance, utilizing two-stage feature ranking, and applying hyperparameter optimization techniques. Notably, our model achieves a remarkable classification accuracy of up to 86.5%, validated through rigorous 10-fold cross-validation technique.

In summary, our study addresses critical gaps in existing literature by employing advanced techniques to better understand the environmental factors influencing Ft’s persistence in soil. This, in turn, offers valuable insights for controlling disease outbreaks, ultimately contributing to broader socio-economic well-being. The contributions of this work are given below:

1. Introduction and Dataset: Introduce a unique soil feature dataset for Ft +Ve and −Ve sites, consisting of 21 soil characteristics.
2. Methodology Innovation: Apply machine learning techniques, specifically Bayesian and Random Ssearch optimization, in contrast to traditional approaches, to analyze the behavior of *Francisella tularensis* (Ft) in soil.
3. Feature Ranking Comparison: Evaluate the performance of feature-ranking models against various classifiers on nested subsets of the ranked attributes.
4. Classifier Performance Improvement: Enhance the performance of classifiers through the application of Bayesian and Random Search optimization techniques.
5. Two-Stage Feature Ranking: Implement a two-stage feature-ranking process. Initially, soil attributes are ranked by different feature-ranking approaches. Subsequently, the weighted score of features is calculated to determine the final rank, utilizing a combination of techniques.
6. Hyperparameter Optimization: Perform classification using hyperparameter optimization techniques, achieving a classification accuracy of up to 86.5%.
7. Validation through Cross-Validation: Verify the proposed model’s performance through a rigorous 10-fold cross-validation.

## Material and methods

**Figure 1:**
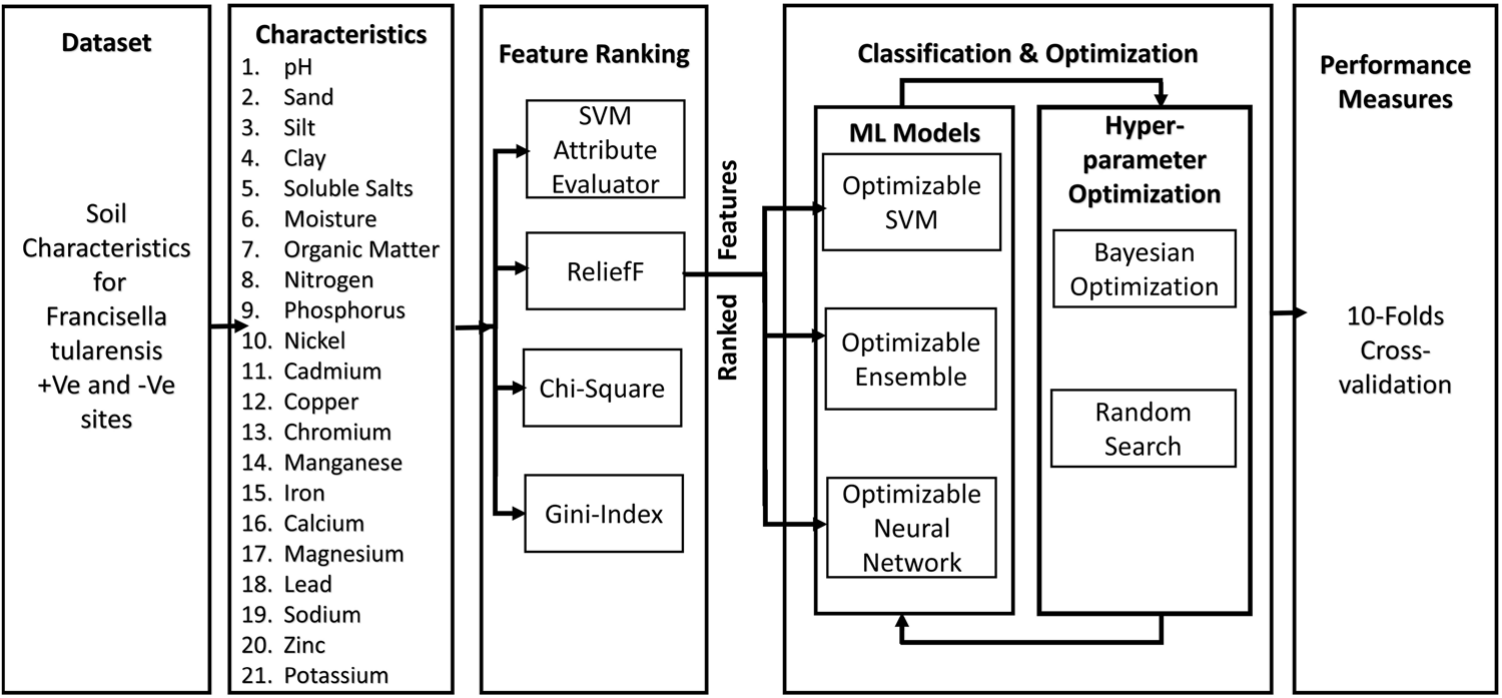
Different stages of *Francisella* *tularensis* feature-ranking, classification and optimization.

The research employs a systematic approach, starting with the ranking of soil features through various techniques such as SVM attribute evaluator, ReliefF, Chi-Square, and Gini-Index algorithms. Following feature ranking, a nested classification methodology is implemented. This involves iteratively selecting the top-ranked features and applying them to optimize classifiers through hyperparameter optimization techniques. The nested classification approach allows for a stepwise refinement of the model, ensuring that the classifiers are tailored to the most relevant features. This sequential strategy, illustrated in The Fig. 1, aims to enhance the robustness and predictive accuracy of the classification model.

### Sample acquisition and analysis

The study was conducted in Punjab province, recognized for its predominant agricultural setting and substantial human and livestock populations. Employing a three-stage sampling design, we selected districts representing key livestock production areas with heightened annual disease incidence. Locations across the provience, including livestock barns and agricultural land, identified as Ft positive, underwent soil chemistry analysis. An equivalent number of locations where Ft genome was not detected were also selected to explore the relationship between soil parameters and bacterial persistence. For soil genome detection, we adhered to a previously optimized and validated real-time PCR protocol targeting the tul4 gene^23^, incorporating necessary controls.

Soil samples were analyzed using optimized protocols for pH, moisture, texture, total soluble salts, and various elements. Detailed methodologies for the analyses can be found in the cited references^24–31^. These physicochemical soil features have different range of values, as displayed in Table 1. The implementation of proper personal protective equipment (PPEs) were ensured during expirementation to maintain biosafety standards. A concise overview of soil sampling, genome extraction, detection, Ft distribution, and soil chemistry analysis is available in our prior research^32^.

**Table 1:** Range of different physicochemical soil characteristics.

Soil characteristics	Range of attributes
1. Cadmium (Cd)	0.03–3.84 mg/kg
2. Calcium (Ca)	40.8–259.9 mg/kg
3. Clay (cy)	1.00–92.0 mg/kg
4. Chromium (Cr)	0.002–0.48 mg/kg
5. Copper (Cu)	0.02–2.36 mg/kg
6. Iron (Fe)	0.34–53.9 mg/kg
7. Lead (Pd)	0.22–7.60 mg/kg
8. Magnesium (Mg)	20.37–324.4 mg/kg
9. Manganese (Mn)	0.09–49.26 mg/kg
10. Moisture (MO)	3.30–15.0%
11. Nickel (Ni)	0.0024–14.43 mg/kg
12. Nitrogen (N)	0.04–0.22 mg/kg
13. Organic Matter (OM)	0.73–4.42 mg/kg
14. Phosphorus (P)	0.36–110.0 mg/kg
15. Potassium (K)	6.70–448.6 mg/kg
16. pH	5.9–12.2
17. Sand	7.00–97.0 mg/kg
18. Silt (Si)	0.00–60.0 mg/kg
19. Sodium (Na)	21.1–304.9 mg/kg
20. Soluble Salts (SS)	0.69–5.04 mg/kg
21. Zinc (Zn)	0.16-1.85 mg/kg

### Appropriate dataset for analysis

To propose a trustworthy and efficient machine learning design, one should select those soil characteristics that are crucial for the growth and survival of Ft. The study identifies the important features, including Soluble Salt (SS), Moisture (MO), pH, Clay (cy), Organic Matter (OM), Silt (Si), Sand, Magnesium (Mg), Phosphorus (P), Nitrogen (N) Copper (Cu), Nickel (Ni), Chromium (Cr), Lead (Pb), Cobalt (Co), Manganese (Mn), Cadmium (Cd), Iron (Fe), Calcium (Ca), Sodium (Na), and Potassium (K), which were utilized for the analysis.

### Attribute selection

Data filtering is important for constructing an accurate and efficient model that can enhance performance. These models assist us in selecting the optimal set of features for analysis. If 21 input attributes are selected from the soil attribute dataset, the attribute matrix, represented by X_em_=[X_1m_, X_2m_, X_3m_,..., X_Em_], consists of E column vectors, and x_em_ is a specific feature value (with e= 1, 2, 3, 4, $\ldots$ E and m= 1, 2, 3, 4, $\ldots$ M; where E=21 and M=148 in the dataset).

### Attribute selection models

A feature selection algorithm incorporates a search procedure to recommend new feature subsets with evaluation criteria that assign different scores to various features^33^. The most appropriate model is the one that tries every likely subset of features and uncovers the most suitable subset that decreases the error rate. Yet, the exhaustive search technique becomes unviable in more comprehensive feature space scenarios. The selection of evaluation metrics greatly influences the procedure. Various feature-selection models have been employed, for example, Support Vector Machine (SVM) attribute evaluator, ReliefF (RLF), Chi-Square (Chi-Sq) and Gini-Index (GI). The feature ranking models are explained as under:

#### SVM attribute evaluator

This attribute evaluator assesses a feature’s worth by using SVM. The features are ranked by the SVM’s square of the weights approach. Feature ranking for multiclass scenarios is managed by ranking each class separately, employing a one-vs-all approach, and then dealing from the top of each pile to suggest a final rank.

#### ReliefF

The ranking model’s main idea is to assess the attributes’ quality by their capability to differentiate among samples of different classes in a local neighborhood. So the most relevant attributes are those that contribute more to increasing the distance between different class samples while contributing less to increasing the distance between the same class samples^34^. The equation for weight updation using RLF is shown as under:

1 $$\begin{aligned} W_{z}=W_{z}-\frac{{\text {diff}}(Z, E, C_h)^{2}}{n}+\frac{{\text {diff}}(Z, E, C_m)^{2}}{n} \end{aligned}$$

Where $W_z$ represents the weight for attribute *Z*, *E* is a randomly sampled instance, $C_h$ and $C_m$ represent the closest hit and closest miss, respectively, and *n* is the number of randomly sampled instances. The diff() function calculates the difference between two instances for a given attribute. For nominal attributes, it is defined as 0 if the values are the same and 1 if the values are different. For continuous features, the actual difference is normalized to the interval 0,1. Dividing the formula by *n* ensures the weights are within the interval -1,1. RLF is sensitive to attribute interactions and aims to estimate the change in probability for the weight of feature Z as defined in equation (2).

2 $$\begin{aligned} W_z= & {} P\left( \frac{different\; value\; of\; Z }{ closest\;example \;of\; different \;class}\right) \nonumber \\{} & {} -P\left( \frac{different\; value\; of\; Z }{ closest \;example\; of\; same \;class}\right) \end{aligned}$$

3 $$\begin{aligned} ReliefF_Z= & {} P\left( \frac{different\; value\; of\; Z }{ different\; class}\right) \nonumber \\{} & {} -P\left( \frac{different \;value\; of\; Z }{ same\; class}\right) \end{aligned}$$

#### Chi-square

Chi-Square (Chi-Sq) is employed for categorical attributes in a dataset. We calculate Chi-Sq between each feature and the target class and pick the expected number of attributes with the best Chi-Sq scores. A high score reveals that the corresponding feature is essential. The technique decides if the sample’s relationship between two categorical variables would reflect their natural association in the population. The Chi-Sq score is shown as follows :

4 $$\begin{aligned} \chi _e^2=\sum \frac{\left( OF_i-EF_i\right) ^2}{EF_i} \end{aligned}$$

Where *e* represents degree of freedom, *OF* (Observed frequency) is the number of instances of a class, *EF* (Expected frequency) if the number of expected instances of class if there is no association betweeen the targer and attribute.

#### Gini-index

Gini-Index (GI), called Gini impurity, estimates the probability of a particular attribute being misclassified when picked randomly. It can be called pure if all the components are associated with a single class. GI ranges between values one and zero, where zero represents the purity of classification, i.e., all the components represent a specific class or only one class exists. Moreover, 1 demonstrates the random distribution of components across different classes. However, 0.5 displays an equal distribution of components over distinct classes. The GI is calculated by subtracting the aggregate of the squared probabilities of a class from 1. The GI can be represented as follows:

5 $$\begin{aligned} \text{ Gini } \text{ Index } =1-\sum _{a=1}^n\left( P_a\right) ^2 \end{aligned}$$

Where $P_a$ exemplifies the likelihood of an element that is classified for a distinct class.

### Hyperparameter optimization

Model optimization is one of the toughest challenges in implementing machine learning solutions. Finding appropriate hyperparameters is crucial for models. However, setting these hyperparameters to achieve good results takes time and effort. There are often general rules of thumb or heuristics for configuring hyperparameters. A better technique is to search various values for a model’s hyperparameters and choose a subset that achieves the best performance on a given dataset. This approach is called hyperparameter tuning or hyperparameter optimization. In contrast to model parameters, the ML engineer sets hyperparameters before training. The weights in a NN are model parameters learned during training, and the number of trees in a random forest is a hyperparameter. They are the configuration settings to be adjusted so that the model can resolve a machine-learning problem optimally. Some of the hyperparameter optimization techniques which were used during experimentation are:

#### Random Search optimization

Random Search (RS) is a family of numerical optimization techniques that do not need the gradient of the problem to be optimized. RS can be employed on procedures that are not differentiable or continuous. Such optimization approaches are derivative-free, black-box, or direct-search methods. RS belongs to the areas of Global Optimization and Stochastic Optimization. It is a direct search approach as it does not need derivatives to explore a continuous domain. This approach relates to minor improvement strategies, such as Adaptive Random Search and Directed Random Search.

#### Bayesian optimization

Bayesian optimization (BO) is a well-known technique for hyperparameter optimization of classifiers. A hyperparameter is an internal parameter of a classification algorithm, like an ensemble classifier’s learning rate or an SVM model’s box constraint. These settings can enormously impact a classifier’s performance, but optimizing them is generally challenging or time-consuming. Typically, optimizing hyperparameters means trying to minimize a classifier’s cross-validation loss. BO locates a point that minimizes the objective function. Suppose we have a function $f:\mathscr {Y} \rightarrow \mathbb {R}$ that we wish to minimize on some domain $Y \subseteq \mathscr {Y}$ . That is, we wish to find

6 $$\begin{aligned} y^*=\underset{y \in Y}{\arg \min }\ f(y) \end{aligned}$$

This problem is generally known as global optimization. The function can be stochastic or deterministic, meaning it can return different results when evaluated at the same point. An revolution in BO is the acquisition procedure, which the technique employs to choose the successive points to assess. The acquisition procedure can stabilize sampling at positions with low-modeled objective functions and explore areas that still need to be modeled well. The Optimization function internally retains a Gaussian process (GP) that uses the objective procedure estimations to train the model. The GP equation is given as under:

7 $$\begin{aligned} p(f)=\mathscr {G} \mathscr {P}(f ; \mu , K) \end{aligned}$$

Given observations $\mathscr {D}=(\textbf{Y}, \textbf{f})$ we can condition our distribution on $\mathscr {D}$ as usual:

8 $$\begin{aligned} p(f \mid \mathscr {D})=\mathscr {G} \mathscr {P}\left( f ; \mu _{f \mid \mathscr {D}}, K_{f \mid \mathscr {D}}\right) \end{aligned}$$

How do we pick where to observe the function next for a given set of observations? A strategy in BO is to devise an acquisition function a(y). It is a cost-effective estimate calculated at a particular point, based on the anticipated benefit of evaluating *f* at *y* in the minimization problem. The optimization of the acquisition function is used to determine the location of the next observation. In essence, we have substituted the original optimization problem with another optimization problem, but one that operates on a much cheaper function a(y).

### Machine learning classifiers

In this section, we outline the various machine learning classifiers utilized in our study, including Support Vector Machine (SVM), Ensemble model (EM), and Neural Networks (NN) for training the proposed model.

#### SVM

SVM performs multi-class classification tasks by drawing a hyperplane to maximize the margin among classes. The classifier also tries to minimize the error^35^, and it provides different advantages like a sufficient generalization to the new instances, the absence of local minimums, and a representation that relies on a few features^36^. Given a training set of input vectors $\textbf{x}_{i} \in R^{d}$, $i=\{1,\ldots ,N_{t}\}$ for d dimensional input space and outputs $y_{i} \in \{1,-1\}$. Where equation 9 shows the SVM’s hyperplane:

9 $$\begin{aligned} y_{i}&= sign(\textbf{w} \cdot \textbf{xy}^T_{i} + b) \end{aligned}$$

In the above equation, $\textbf{x}$ describes the input vector, and $\textbf{w}$ is for a constant vector of an SVM hyperplane. While the training input vector $\textbf{x}_{i}$ illustrates the attributes and *sign*() is a signum function with ±1 output. The goal is to minimize Equation 10.

10 $$\begin{aligned}&\min _{w,b,\zeta }\text { } \frac{1}{2} ||\textbf{w}||^{2}+C_{b}\sum \zeta _{i}\\ \text { (subject to) } \text { }&y_{i} (\textbf{w}^T\textbf{x}_{i}+b) \ge 1-\zeta _{i}&(\forall i) \nonumber \\&\zeta _{i} \ge 0&(\forall i) \nonumber \end{aligned}$$

Where $C_{b}$ represents the box constraint and $\zeta _{i}$ disciplines objective function for samples that cross a specific margin that signifies a particular class.

#### Ensemble model

An ensemble is a predictive method that comprises a weighted combination of numerous classification models. In general, fusing numerous classification models improves the performance enormously.

#### Neural networks

A NN comprises a feed-forward and backpropagation network, which includes three types of layers: an input layer, an output layer, and a hidden layer. Each layer in the network has a specific role to play. The input layer accepts the input data, while the output layer carries out key functions such as prediction and classification. The hidden layers are the true workhorse of the model, executing the majority of the computation between the input and output layers. The backpropagation technique optimizes the weights of these layers. These models are used for classification, recognition, approximation, and prediction tasks and are effective for solving non-linearly separable problems. The computations taking place at each neuron in the hidden and output layer are as under:

11 $$\begin{aligned} \textrm{O}(\textrm{z})= & {} \mathrm {r_2}(\textrm{A}(2)+\textrm{W}(2) \textrm{h}(\textrm{z})) \end{aligned}$$

12 $$\begin{aligned} \textrm{h}(\textrm{z})= & {} \Phi (\textrm{z})=\mathrm {r_1}(\textrm{B}(1)+\textrm{W}(1) \textrm{z}) \end{aligned}$$

Let *W*(1), *W*(2) represent the weights and *B*(1), *B*(2) be the biases of the previous and next layer. The output of the previous layer, *z*, is multiplied with the weights of the current layer, *W*(1), to form the inner vector product. Then, a bias vector *B*(1) is added, and the result is fed into the activation function $r_1()$. The activation functions ${r_1, r_2}$ are used to introduce non-linearity into the model. The various activation functions are $\{r_1, r_2\}$. The mostly applied activation functions are sigmoid and tanh , where sigmoid is shown as ${\text {sigmoid}}(d)=1 /\left( 1+e^{-d}\right)$ and tanh is $\tanh (d)=\left( e^{d}-e^{-d}\right) /\left( e^{d}+e^{-d}\right)$.

## Experiments

### Data description

All the feature-ranking and hyperparameter optimization experimentations on machine learning models are performed on the *F. tularensis* soil attribute dataset, comprising 148 samples. Each sample consists of 21 soil features. A supervised dataset is required to prepare a predictive model for classification. So, we assigned label “A” to positive samples, and “B” to negative soil samples in the dataset.

### Software tool and performance measures

We use MATLAB for experimentation on the Ft soil attribute dataset for hyperparameter optimization of classification models and feature ranking. Initially, we load the dataset to the workspace, and then a 10-folds validation scheme is applied, which measure’s a model’s accuracy. Once the app has loaded the data, we can choose from several feature selection algorithms available in MATLAB for feature ranking. Next, we choose models that can be optimized for accuracy calculation by picking the top-ranked features from the dataset sequentially using the nested subset method. These models adjust their parameters automatically by testing various hyperparameter combinations through an optimization process. The objective of this process is to minimize classification errors or costs. The accuracy of the model can be viewed in the history panel, and its classification errors can be seen by clicking on the confusion matrix icon in the plot section.

### Hyper-parameters for classifiers

In this section, we outline the key hyperparameters employed for the classifiers, including Support Vector Machines (SVM), Ensemble models, and Neural Networks, during the experimental phase.

#### SVM implementation

The MATLAB implementation of the SVM model underwent comprehensive parameter optimization to enhance overall performance. The key parameters considered included the box-constraint level, kernel scale, data standardization, multiclass function, and kernel type. The box-constraint level, influencing the balance between smooth decision boundaries and accurate classification of training points, was fine-tuned to 780 in the optimized SVM model. A Gaussian kernel was specifically chosen to shape the decision boundary, with the kernel scale meticulously set to 16.3794 for optimal performance. Various kernel functions, including Gaussian, Linear, Quadratic, and Cubic, were explored. Data standardization was implemented to ensure consistency in input feature scaling. The multiclass function, offering the choice between One-vs-All or One-vs-One, was tailored to a one-vs-one configuration for multi-class scenarios. These optimizations aimed to strike a balance in decision boundary smoothness and accurate classification, with the chosen configurations contributing to the robustness of the SVM model.

#### Ensemble model implementation

The implementation of the Ensemble model in MATLAB underwent a thorough optimization process for key parameters, each playing a crucial role in shaping the model’s overall performance. The *number of learners*, pivotal for balancing complexity and computational efficiency, was optimized within the range of 10–500, ultimately set to 22. The *maximum number of splits*, ranging from 1 to 147, was meticulously tuned to 4, enhancing the model’s capacity to capture intricate dataset relationships. Similarly, the *number of predictors to sample* underwent optimization within the range of 1–14, with the final value set to 14, striking a balance between diversity and efficiency during the learning process. The *learning rate*, critical for optimization convergence, was fine-tuned within the range of 0.001–1, with the optimized value set to 0.95019. Various ensemble types, including AdaBoost, RUSBoost, LogitBoost, GentleBoost, and Bag, were explored, with AdaBoost yielding the most effective results. This comprehensive parameter configuration ensures the robustness and optimal predictive capabilities of the Ensemble model.

#### Neural network implementation

The implementation of the neural network in MATLAB involved the optimization of several key hyperparameters, each exerting a significant impact on the overall performance of the model. The number of fully connected layers, ranging from 1 to 3, was explored, with the optimal configuration determined as two layers. The size of each layer, including the first, second, and third layers, varied between 1 and 300. For optimal results, the number of neurons in the first layer is set to one, and in the second layer is set to two. The regularization strength (Lambda) played a crucial role, with a range from 6.7568 e−08 to 675.6757, and the optimized value was set to 0.01174. Data standardization, configurable as either true or false, was implemented to ensure consistency in the scale of input features, contributing to the robustness of the neural network model. Activation functions, including ReLU, Tanh, and Sigmoid, were explored, with the Tanh function identified as the most effective. These meticulous configurations collectively aimed to achieve optimal performance and reliability in the neural network model.

## Results

This section presents the outcomes of attribute-ranking methods and their comparison to classifiers optimized through hyperparameter optimization techniques. Bayesian and random optimizations, along with cross-validation, are applied to SVM, Ensemble, and Neural Networks to enhance performance and mitigate overfitting.

Initially, four attribute-ranking techniques are employed for the Ft dataset. Table 2 outlines rankings for various attribute-ranking models: ReliefF (RLF), SVM, Chi-Sq, and GI. The “Attribute Index” column assigns a unique value to each soil feature, with pH indexed as 1, sand (Sd) as 2, silt (Si) as 3, and so forth. The first row in columns rk(ReliefF), rk(SVM), rk(Chi-Square), and rk(Gini-Index) designates the top-ranked attribute, which is 4 (Cy). The second row lists the subsequent best-ranked features, namely 8 (N), 18 (Pb), 3 (Si), and 8 (N), respectively. Similarly, the final row displays the least of the best-ranked features: 21 (K), 15 (Fe), 12 (Cu), 21 (K). Furthermore, when we examine the top 10 attributes from all the attribute-ranking models in Table 2, we can draw the following conclusions:

1. Five attributes-Zinc (Zn), Clay (Cy), Soluble Salts (SS), Nitrogen (N), and Silt (Si)-appear consistently across all feature-ranking models.
2. Six attributes-Zinc (Zn), Clay (Cy), Soluble Salts (SS), Nitrogen (N), Silt (Si), and Lead (Pb)-are present in the rankings of SVM, Chi-Square (Chi-Sq), and Gini-Index (GI).
3. Seven attributes-Nickel (Ni), Zinc (Zn), Clay (Cy), Soluble Salts (SS), Nitrogen (N), Silt (Si), and Moisture (Ms)-are shared between ReliefF (RLF) and SVM.
4. Another set of seven attributes-Magnesium (Mg), Zinc (Zn), Clay (Cy), Soluble Salts (SS), Nitrogen (N), Silt (Si), and Organic Matter (OM)-are common among ReliefF (RLF), Chi-Square (Chi-Sq), and Gini-Index (GI).
5. Finally, nine attributes-Magnesium (Mg), Manganese (Mn), Zinc (Zn), Clay (Cy), Soluble Salts (SS), Nitrogen (N), Silt (Si), Lead (Pb), and Organic Matter (OM)-are shared between Chi-Square (Chi-Sq) and Gini-Index (GI).

Similarly, as shown in Table 2, when examining the 11 attributes contributing the least, five of them -Potassium (K), Calcium (Ca), Chromium (Cr), Copper (Cu), and pH- persist across all feature-ranking models.

**Table 2:** Attribute-ranking for Ft in soil using various attribute selection methods.

Featureindex	Soilfeatures	rk(ReliefF)	rk(SVM)	rk(Chi-Square)	rk(Gini-Index)
1	pH	4	4	4	4
2	Sand (Sd)	8	18	3	8
3	Silt (Si)	5	5	8	7
4	Clay (Cy)	7	8	14	14
5	Soluble Salts (SS)	20	2	5	3
6	Moisture (Ms)	17	20	18	18
7	Organic Matter (OM)	6	3	7	5
8	Nitrogen (N)	19	10	17	20
9	Phosphorus (P)	3	9	15	17
10	Nickel (Ni)	10	6	20	11
11	Cadmium (Cd)	1	14	1	6
12	Copper (Cu)	12	11	13	10
13	Chromium (Cr)	14	7	11	15
14	Manganese (Mn)	11	21	10	13
15	Iron (Fe)	2	13	9	9
16	Calcium (Ca)	13	1	16	2
17	Magnesium (Mg)	18	17	6	1
18	Lead (Pb)	16	12	2	19
19	Sodium (Na)	15	19	19	16
20	Zinc (Zn)	9	16	21	12
21	Potassium (K)	21	15	12	21

**Figure 2:**
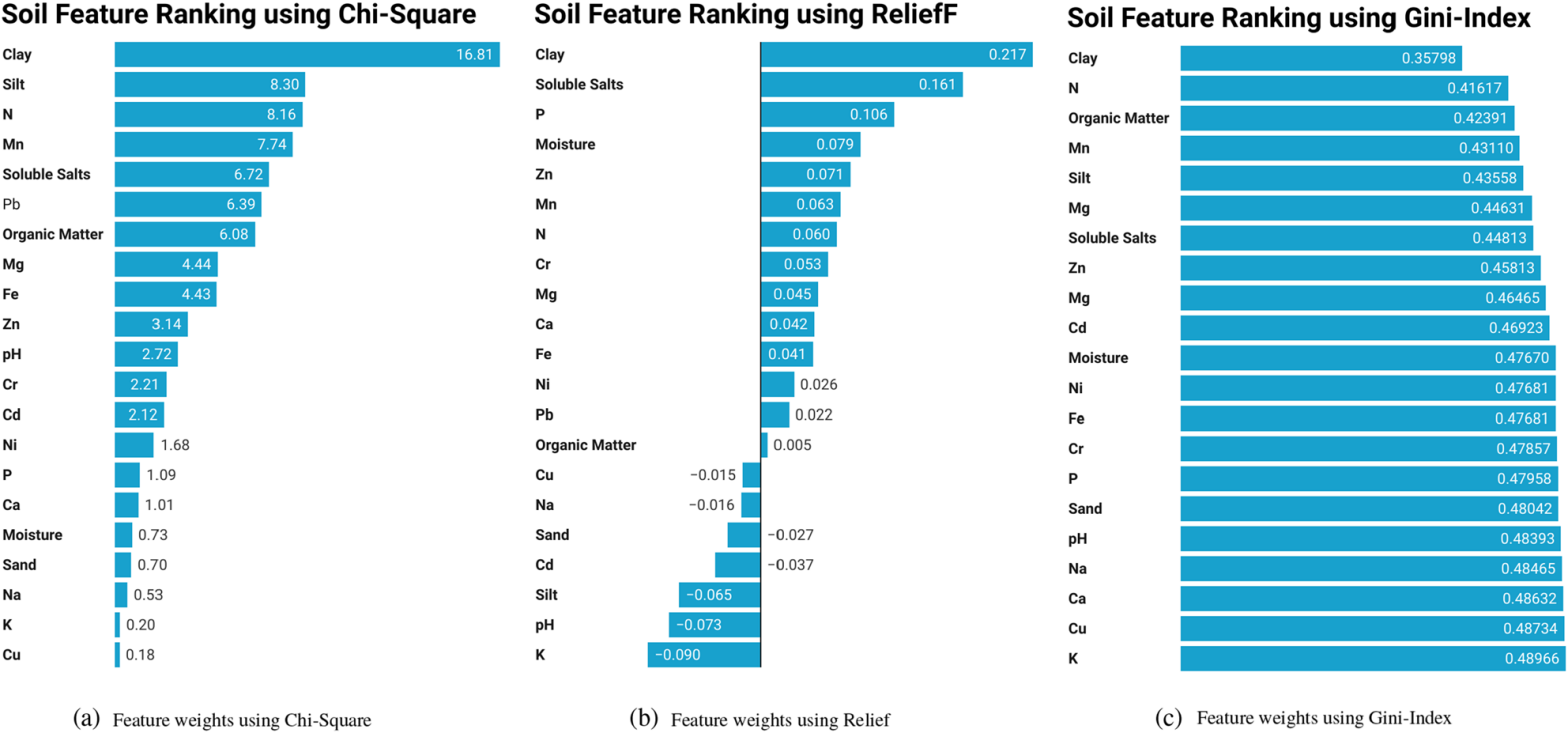
Feature weight for different ranking alogrithmns.

The Fig 2 illustrates the outcomes of three distinct feature ranking algorithms: Chi-Square, ReliefF, and Gini-Index. In the Chi-Square algorithm, Clay emerges as the most influential feature with a substantial weight of 16.81. Silt and Nitrogen follow closely with weights of 8.30 and 8.16, emphasizing their significant contributions to the classification. Conversely, Copper and Potassium are identified as the least significant features, each receiving minimal weights of 0.18 and 0.20. The ReliefF algorithm corroborates the significance of Clay, ranking it as the most important soil feature with a weight of 0.217. Following Clay, Soluble Salts and Phosphorus exhibit weights of 0.161 and 0.106, respectively. Notably, Potassium and pH emerge as the least significant features with weights of $-0.090$ and $-0.073$. Similarly, the Gini-Index algorithm underscores Clay as the most crucial feature, assigned a weight of 0.35798. Nitrogen and Organic Matter follow closely with weights of 0.41617 and 0.42391, respectively. On the other hand, Potassium and Copper are identified as the least significant features, each with weights of 0.48966 and 0.48734. These weights offer a quantitative measure of each feature’s impact, facilitating the identification of key contributors and less influential variables in the context of pathogen prevalence in soil.

Next, we perform a two-stage attribute ranking to assess each feature’s impact on the prevalence of Ft in soil-related environments. Initially, various feature-ranking approaches are employed to rank soil features, followed by the calculation of weighted scores to determine the final rank using a combination of techniques. Tables 3 and 4 showcase the top-ranked and least-ranked soil features, respectively. These tables present the scores assigned by each attribute-ranking model and the cumulative score for each feature in the Ft soil feature dataset. The final score represents the sum of scores from all feature-ranking models. A lower score indicates a higher rank, while a higher score implies a lower rank for the soil attribute.

The 1*st* row of the Table 3 shows that Clay (Cy) holds the 1st rank in RLF, SVM, Chi-Sq, and GI, with a cumulative score of 4 (1+1+1+1=4). The 2*nd* row shows Nitrogen (N) with ranks 2, 4, 3, and 2 by RLF, SVM, Chi-Sq, and GI, respectively, resulting in a cumulative score of 11. Similarly, the last row indicates Nickel (Ni) ranked 10, 8, 14, and 12 by RLF, SVM, Chi-Sq, and GI, respectively, with a cumulative score of 44. Clay (Cy) emerges as the top-ranked feature with a cumulative score of 4, while Nitrogen (N) secures the 2nd position with a cumulative score of 11. Similarly, the last row indicates that Nickel (Ni) holds the 10th rank, accumulating a cumulative score of 44. Likewise, examining the details in Table 4 reveals that Potassium (K) holds the lowest rank, having a cumulative score of 76. This score is obtained by summing the scores from all feature-ranking models ($21+14+20+21=76$). Following closely behind are Calcium (Ca), Copper (Cu), and Sodium (Na), with cumulative scores of 73 ($18+20+16+19$), 71 ($12+18+21+20$), and 64 ($8+19+19+18$), respectively, and so forth.

**Table 3:** Index of best-ranked features for Francisella in soil.

Top ranked attributes	rk(ReliefF)	rk(SVM)	rk(Chi-Square)	rk(Gini-Index)	Ranking Score of each Attribute
Clay (Cy)	1	1	1	1	4
Nirogen (N)	2	4	3	2	11
Soluble salts (SS)	3	3	5	7	18
Silt (Si)	9	7	2	5	23
Organic matter (OM)	4	13	7	3	27
Zinc (Zn)	5	6	10	8	29
Lead (Pb)	17	2	6	6	31
Manganese (Mn)	13	11	4	4	32
Magnesium (Mg)	6	17	8	9	40
Nickel (Ni)	10	8	14	12	44

**Table 4:** Index list of least-ranked features for Francisella in soil.

Least ranked attributes	rk(ReliefF)	rk(SVM)	rk(Chi-Square)	rk(Gini-Index)	Ranking score of each attribute
Potassium (K)	21	14	20	21	76
Calcium (Ca)	18	20	16	19	73
Copper (Cu)	12	18	21	20	71
Sodium (Na)	8	19	19	18	64
Iron (Fe)	19	21	9	13	62
Phosphorus (P)	20	9	15	15	59
Chromium (Cr)	16	15	12	14	57
pH	11	16	11	17	55
Sand (Sd)	15	5	18	16	54
Cadmium (Cd)	14	12	13	10	49
Moisture (Ms)	7	10	17	11	45

**Figure 3:**
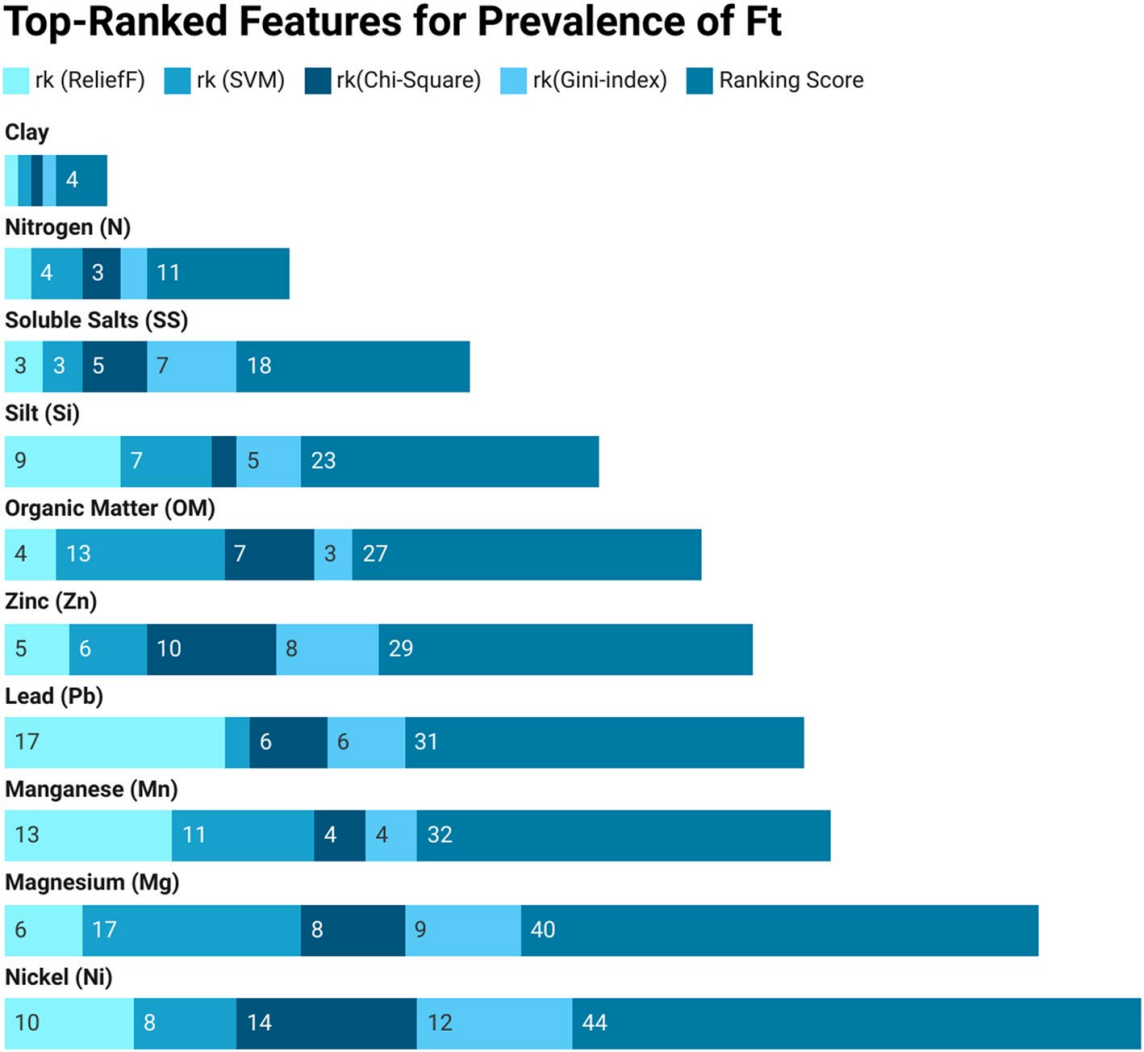
Best-ranked features for Francisella in soil.

**Figure 4:**
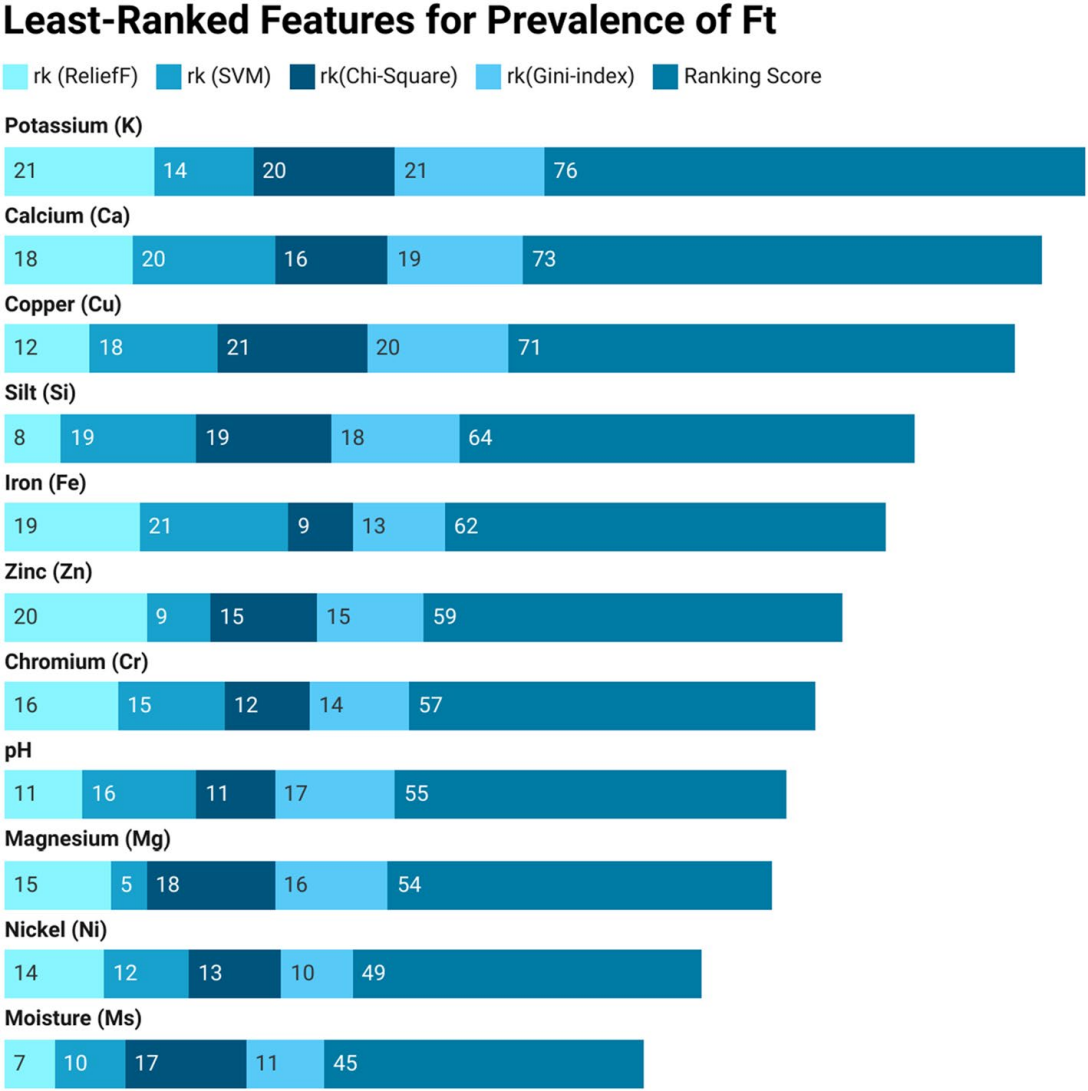
Least-ranked features for Francisella in soil.

The bar charts in Figs. 3 and 4 offer a clear overview of attribute rankings, presenting the cumulative score for each feature in distinct colors. Different shades of blue represent the ranking scores (rk) for ReliefF (RLF), Support Vector Machine (SVM), Chi-Square (Chi-Sq), and Gini-Index (GI), while the dark blue “Ranking Score” indicates the cumulative score across all feature-ranking methods. The best-ranked attribute, Clay (Cy), secures the top position with a cumulative score of 4. Various shades of light blue represent the ranking scores from different methods, all of which are 1 for each algorithm. The final cumulative score, depicted in dark blue, is achieved by combining the rankings across all feature-ranking methods ($1+1+1+1=4$), and so on. Similarly, for the least-ranked attribute, Potassium (K), claims the lowest position with a cumulative score of 76. Distinct light blue shades represent scores from different methods-21 for RLF, 14 for SVM, 20 for Chi-Sq, and 21 for GI. The final cumulative score, represented in dark blue, is obtained by summing the rankings across all feature-ranking methods (21+14+20+21=76), and so on.

Next, we evaluated the performance of various attribute-ranking models against different classifiers, optimizing them using Bayesian and random search techniques for improved results. The experimental outcomes are presented in Table 5. For ReliefF (RLF), the “rank” row indicates the sequence of ranked features. The table then showcases the results of Bayesian and random search optimization for various machine learning classifiers (SVM, EM, and NN) based on the RLF ranking. Classification accuracy ranges from 86.5 (SVM) to 73.6% (NN) with different ranking models, classifiers, and optimization techniques.

**Table 5:** A Comparative analysis of for different optimization techniques against different Machine learning classifiers using ReliefF attribute selection method.

		Subset																				
Ranker	Classifier	1	2	3	4	5	6	7	8	9	10	11	12	13	14	15	16	17	18	19	20	21
ReliefF	Rank	4	8	5	7	20	17	6	19	3	10	1	12	14	11	2	13	18	16	15	9	21
	SVM	77	75.5	82.4	81.1	81.1	81.1	79.7	80.4	79.1	79.1	79.1	79.7	82.4	82.4	86.5	83.1	83.8	85.1	79.9	82.4	83.1
Bayesian	Ensemble	75	77.7	79.9	76.4	80.4	78.4	77.7	78.4	80.4	77.7	81.1	80.4	81.1	81.1	79.7	79.1	81.8	81.1	81.8	78.4	79.9
Optimization	Neural network	75	76.4	81.1	83.8	77.7	81.1	80.4	81.1	79.1	79.7	77	79.1	81.8	79.1	77.7	83.8	78.4	80.4	81.1	79.7	81.1
	SVM	73.6	77.7	81.1	79.7	79.7	81.1	81.1	81.8	79.1	79.1	77.7	77	81.8	82.4	86.5	83.1	83.8	84.5	80.4	82.4	82.4
Random	Ensemble	77	77.7	79.9	81.1	80.4	79.1	79.1	77.7	79.7	74.4	74.3	77	81.1	81.1	81.1	80.4	79.7	76.4	77.7	79.7	81.1
Search optimization	Neural network	73	77	77.7	79.9	78.4	79.7	77.7	75.7	79.7	79.1	79.1	80.4	79.9	79.9	83.1	79.1	77.7	81.8	82.4	78.4	79.7

The attribute with the most impact for RLF is Cy. Using this attribute, SVM, EM, and NN achieve accuracies of 77%, 75%, and 75%, respectively, and 73.6%, 77%, and 73% for Bayesian optimization (BO) and Random Search optimization (RS). The results in Table 5 reveal several key findings:

1. The two optimization techniques yield different results for various classification models.
2. For both optimization techniques, SVM achieves an accuracy of 86.5% for 15 soil features.
3. The performance of different classification models is inherently arbitrary:
  - (BO+SVM, 86.5%)
  - (RS+SVM, 86.5%)
  - (BO+EM, 81.8%)
  - (RS+EM, 81.1%)
  - (BO+NN, 83.8%)
  - (RS+NN, 83.1%).
4. The results suggest that the BO optimization technique yields more favorable outcomes for classifiers like SVM, EM, and NN compared to RS.
5. SVM outperforms other classifiers for both BO and RS.
6. BO+SVM produces the best classification results for the 15 soil features: Cy, N, SS, Si, OM, Zn, Pb, Mn, Mg, Ni, Ms, Cd, Si, pH, Cr.
7. Other models, such as BO+NN and RS+NN, also generate noteworthy results of 83.8% and 83.1%, utilizing 16 and 15 soil features, respectively.

Finally, we present our proposed SVM classifier, which was optimized using bayesian optimization technique to generate F-1 Score of 86.5% and accuracy of 86.5%. The details of training results, models details, optimized hyperparameters, and optimizer options are shown in the Table 6.

**Figure 5:**
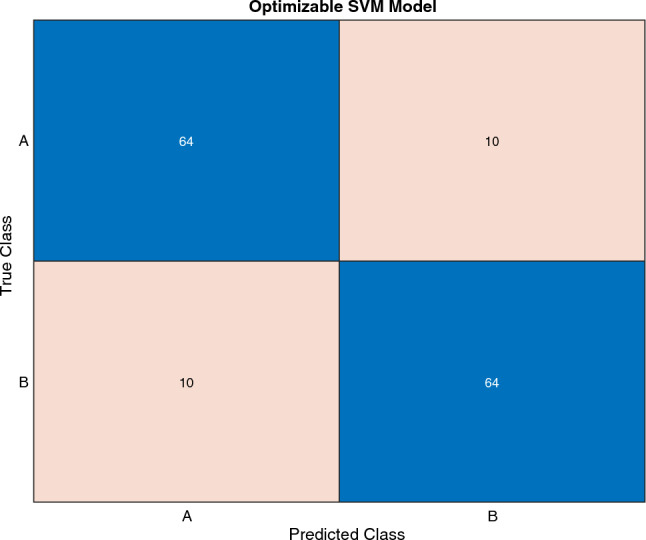
Confusion matrix for proposed SVM model for Ft classification.

**Figure 6:**
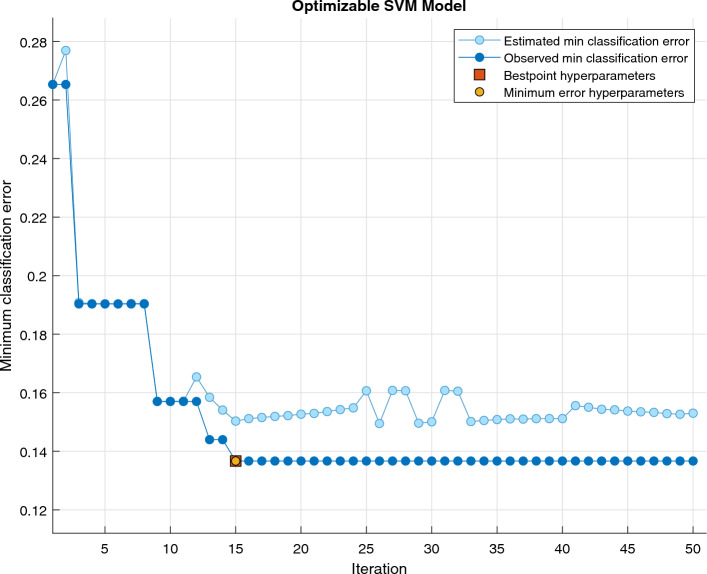
Bayesian optimization error plot for proposed model.

The Fig. 5 depicts the confusion matrix, assessing the performance of the optimized SVM classifier in distinguishing between Class A (Positive) and Class B (Negative). The matrix involves a total of 148 instances, evenly distributed between the positive and negative classes, each comprising 74 instances. Among the 74 positive instances, 64 are correctly classified (True Positives—TP) as Class A, while 10 instances are misclassified (False Negatives—FN) as Class B. Similarly, out of the 74 negative samples, 64 instances are correctly classified (True Negatives—TN) as Class B, with 10 instances being misclassified (False Positives—FP) as Class A. A good classifier has a dominantly diagonal confusion matrix since most of the predictor variables matched the actual labels with only a few off-diagonal numbers that indicate confusion between classes, as is visible in the case of our presented optimized SVM model. The Fig. 6 error plot for the SVM model provides a visual representation of the classification error analysis. In the plot, the estimated minimum classification error is depicted by light blue circler points, while the observed minimum classification error is represented in dark blue points. The orange box highlights the hyperparameters associated with the best-performing point, indicating the configuration that yielded optimal results during the training process. Additionally, the yellow circle signifies the hyperparameters corresponding to the minimum observed error, pinpointing the configuration where the SVM model achieved its highest accuracy. his graphical representation aids in identifying the effectiveness of different hyperparameter settings, allowing for a nuanced understanding of the model’s performance and guiding the selection of optimal configurations for future experiments.

**Table 6:** Details of Results, Optimized hyperparameters, and optimizer for proposed SVM model.

Training results	F-1 Score (Validation)	86.50%
	Accuracy (Validation)	86.50%
	Validation cost	20
	Speed of prediction	$\sim$2500 obs/sec
	Time to train	56.148 s
Model type	Preset: OptimizableSVM	Kernel type: Gaussian
	Box-constraint level: 780	Multiclass function: One-vs-One
Optimized Hyperparameters	Kernel scale:16.3794	Data standardization: true
Optimization Types	Optimization: Bayesian	Acquisition method: Expected improvement per second plus
	No of iterations:50	Time limitation for training: false

**Figure 7:**
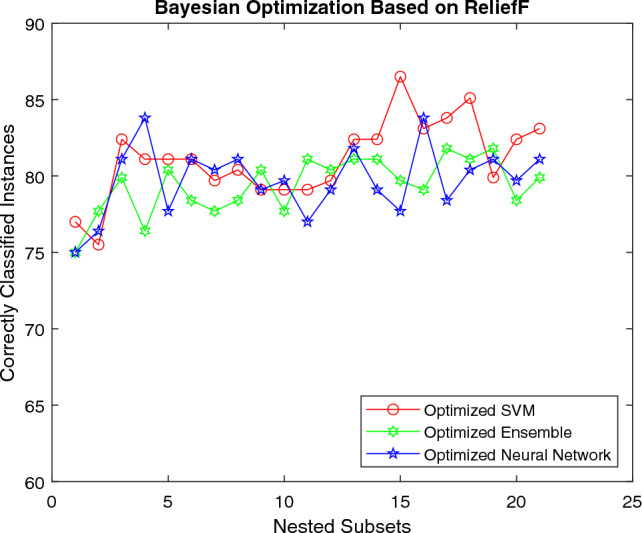
Performance of different classifiers using Bayesian Optimization.

**Figure 8:**
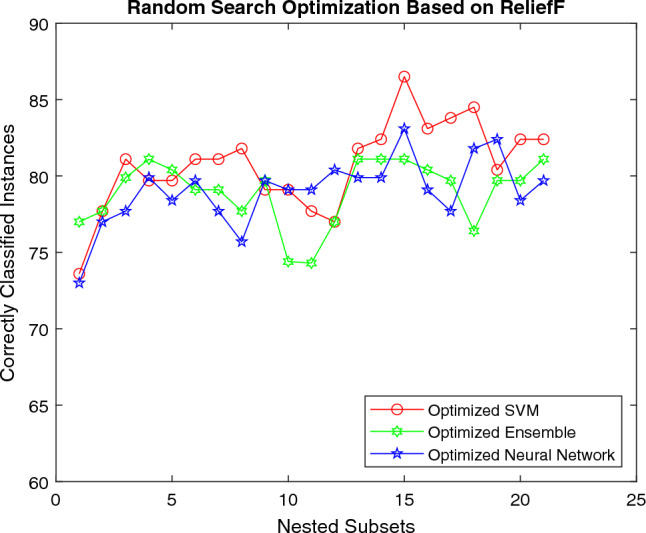
Performance of different classifiers using random search optimization.

The Figs. 7 and 8 exhibit the change in the classification performance of algorithms as the number of attributes is altered while using different hyperparameter optimization techniques. Figure 7 displays the performance of classifiers using RLF and BO strategies. For the same feature set, NN generates more promising results than other classification models for the initial set of features. However, these models show similar results for mid-level features. SVM surpasses other models for the last few attributes. The outcomes illustrate that overall SVM yields the best results by generating an accuracy of 86.5%. So, the overall performance of SVM is far better than other machine learning classifiers

The Fig. 8 shows the accuracy of machine learning models for RLF using RS technique. For the initial set of features all the machine classifiers seem to generate similar resutls better results. However, SVM surpasses all the classification models for mid and final-level features by generating a classification accuracy of 86.5%.

In summary, the results propose that:

1. While assessing the top 10 features, the 5 most contributing features common among all are {Cy, N, SS, Si, Zn}.
2. The 5 least significant features for Ft are { K, Ca, Cr, Cu, pH}.
3. Hyperparameter optimization using BO produces better outcomes than other optimization techniques.
4. SVM is the best performer among classification models.
5. SVM achieves the best classification accuracy of 86.5% for the first 15 soil features {Cy, N, SS, Si, OM, Zn, Pb, Mn, Mg, Ni, Ms, Cd, Si, pH, Cr} using BO and RS.
6. For multi-dimensional data, optimizing the parameters of machine learning models can significantly improve performance by using hyperparameter optimization techniques. Therefore, the selection of correct hyperparameters is essential for yielding good classification results.

### Comparative analysis with prior machine learning techniques

Few recent works applied machine learning for classifying various soil-borne pathogenic bacteria like *F. tularensis* and *C. burnetiia*; and the conditions that support their sustenance in soil, as exhibited in Table  7. But, our presented design uses hyperparameter tuning with two-stage attribute-ranking on a new *F. tularensis* dataset, contrary to previous research.

**Table 7:** A comparative analysis with prior machine learning techniques.

Technique	Soil-based	Hyper-parameter	Classification &	Two-phase	Most related	Least related	Accuracy
	Pathogen	Optimization	Feature-ranking	Feature-ranking	Features	Features	
Proposed model	*F. tularensis*	$\checkmark$	$\checkmark$	$\checkmark$	Clay, nitrogen, soluble salts, silt, organic matter, and zinc	Potassium, calcium, copper, sodium, iron, and phosphorus	86.5%
Ahmad et al.^37^	*C. burnetiia*	$\times$	$\checkmark$	$\checkmark$	organic matter, nitrogen, potassium, cadmium, magnesium, and chromium	Clay, phosphorous, manganese, copper, and moisture	82.98%
Ahmad et al.^22^	*F. tularensis*	$\times$	$\checkmark$	$\times$	Organic matter, nitrogen, clay, soluble salts, silt, nickel, and zinc	Iron, phosphorous, potassium, calcium, chromium, sand, and copper	84.35%
shahbaz et al.^21^	*F. tularensis*	$\times$	$\times$	$\times$	$\times$	$\times$	82.61%

### Discussions

Machine learning models are applied as a benchmark in various fields, like, disease diagnosis^38–41^ bio-informatics^42^, medical science^43^, agriculture^44^, and soil classification^45^. Our work reveals that these models, rather than current statistical techniques demonstrate outstanding results for the classification of *F. tularensis* and learning its behavior in soil settings.

The results highlight the significance of specific soil characteristics for the survival of *F. tularensis*, as illustrated in Table 3. Previous analyses have consistently pointed to abiotic factors, such as organic matter, clay, and various micro-nutrients, as primary drivers of bacterial communities in soil^46–49^. Moreover, these factors positively correlate with the prevalence of soil-borne pathogenic bacteria^50–52^. Clay and silt, known for their increased surface area, are suggested to contain a significant amount of organic matter, potentially fostering the existence of bacteria^53^. Recent studies^16,17,37,54^ also emphasize the importance of soil’s physical and chemical properties, including clay, nitrogen, soluble salts, silt, organic matter, zinc, lead, and nickel, for the persistence of *F. tularensis*, *C. burnetii*, and *B. anthracis*.

Our investigation underscores clay as the most influential attribute for the presence of *F. tularensis* in soil, aligning with previous works^16,32,52^. Subsequent crucial attributes contributing to the sustenance of the bacterial pathogen include nitrogen, soluble salts, silt, organic matter, and zinc. Organic matter is established as beneficial for bacterial survival in soil settings^16,51,52^, while nitrogen is crucial for the persistence of pathogens within their hosts^55^. Zinc, soluble salts, organic matter, and nitrogen are identified as related to the survival of *F. tularensis* in the soil^16,32,56^. Zinc, in particular, plays a role in various cellular operations, including metabolism, gene expression, pH regulation, glycolysis, DNA replication, and amino acid synthesis^57^, with excess zinc potentially inducing toxicity^58^. Recent works^32,54^ suggest a positive association between soluble salts and the prevalence of *F. tularensis* and *C. burnetii*. Additionally, studies^56,59^ indicate that organic matter and nitrogen are associated with the prevalence of A. brasilense and C. burnetii.

The remaining contributing features from Table 3 include lead, manganese, magnesium, and nickel. Our results align with studies^16,22,32^ that establish positive correlations between attributes such as manganese, magnesium, lead, and nickel and *F. tularensis* in soil. Organic matter, manganese, and magnesium are associated with B. anthracis, and magnesium is linked to the prevalence of C. burnetii in soil^17^. Magnesium also contributes to bacterial survival during starvation and cold shocks^60^.

Our study also reveals that cadmium, moisture, sand, and pH play intermediary roles. Earlier works^47–49^ stress the importance of pH, soil texture, and soil nutrients for microbial communities. Recent analysis^22^ supports a positive association between *F. tularensis* and cadmium, pH, and moisture in soil environments. Another work^61^ suggests *F. tularensis* is associated with low temperature and moisture, emphasizing the pathogen’s affinity for these conditions. Univariate analysis^54^ shows significant differences among C. burnetii positive and negative soils for pH, nitrogen, magnesium, soluble salts, and organic matter.

Our results indicate that the least contributing soil attributes, as shown in Table 4, include potassium, calcium, copper, sodium, iron, phosphorus, and chromium. This aligns with recent findings^22^ displaying no substantial differences between *F. tularensis* negative and positive sites concerning copper, sand, iron, calcium, phosphorous, chromium, and sodium in the soil. Conversely, *B. anthracis* and *C. burnetii* exhibit positive affinities to copper, chromium, cobalt, cadmium, sodium, iron, calcium, and potassium^17^. Additionally, research^19^ suggests sodium and potassium facilitate *F. tularensis* growth in water and soil. Recent research^54^ shows no substantial differences among Coxiella positive and negative sites related to copper, chromium, iron, and phosphorus in the soil. Analysis^16^ and similar work^32^ indicate that soil features like copper, chromium, phosphorus, iron, sodium, potassium, and calcium do not exhibit any affiliation with *F. tularensis*. Nonetheless, other studies^62^ acknowledge that the aerobic heterotrophic community is sensitive to various nutrients, including zinc, cadmium, chromium, mercury, manganese, nickel, and copper.

Comparing our current findings with our previous publication on *F. tularensis* using machine learning, we observe a slight variation in the sequence of the most significant factors. In the current work, the order of significance is clay, nitrogen, soluble salts, silt, organic matter, and zinc. However, in our previous work, the sequence was clay, nitrogen, organic matter, soluble salts, zinc, and silt. Similarly, when examining the sequence of least significant factors in the current research, we find potassium, calcium, copper, sodium, iron, and phosphorus to have the least impact. In contrast, our earlier work identified potassium, phosphorus, iron, calcium, copper, chromium, and sand as the least influential. The observed shift in sequence can be attributed to the adoption of a more effective ranking methodology in which features are evaluated based on the accumulative weighted score of all methods. This refined approach allowed us to discern a more nuanced order of significance among the key factors influencing the survival of *F. tularensis* in soil. Furthermore, the implementation of hyperparameter optimization played a pivotal role in enhancing accuracy, leading to an improvement of over 2% compared to our previous work. The meticulous fine-tuning of hyperparameters contributed to a more robust and accurate machine learning model, thereby reinforcing the reliability of our current findings.

## Conclusion and future works

In summary, our study delves into the outcomes of various attribute-ranking methods, comparing their rankings across different classifiers optimized with hyperparameter optimization techniques using Ft positive and negative soil datasets. Beyond the specific case study, our findings underscore the significance of key soil features, with clay emerging as the top-ranked attribute, followed by nitrogen, soluble salts, silt, organic matter, and zinc. The application of Bayesian optimization (BO) demonstrates exceptional results in hyperparameter optimization techniques, contributing to the robustness of our models. Specifically, Support Vector Machine (SVM) stands out as the most effective classifier, achieving an impressive accuracy of 86.5% when considering the first 15 soil features {Cy, N, SS, Si, OM, Zn, Pb, Mn, Mg, Ni, Ms, Cd, Si, pH, Cr} with BO and random search (RS). Expanding beyond SVM, our study explores alternative models such as {BO+NN} and {RS+NN}, showcasing noteworthy classification accuracies of 83.8% and 83.1%, respectively. These models, utilizing 16 and 15 soil attributes, offer valuable insights into understanding the contribution of specific soil features to the prevalence of bacterial pathogens in soil-related environments.

While our investigation provides crucial insights into the interplay between soil characteristics and pathogen prevalence, it is essential to acknowledge that the size of our dataset is limited. In subsequent studies, we aim to enhance the robustness of our findings by expanding the geographical scope of our dataset. Specifically, we plan to explore additional districts within Punjab and extend our investigation to encompass other provinces in the country. By doing so, we aspire to gather a more extensive dataset that encapsulates the diversity of soil characteristics across different regions. This geographical expansion will not only contribute to a more comprehensive understanding of the interplay between soil attributes and pathogen prevalence but also facilitate the development of machine learning models that are more adaptable and representative of diverse environmental conditions.

### Supplementary Information

Supplementary Information 1.

Supplementary Information 2.

Supplementary Information 3.

## Data Availability

The corresponding author can be contacted at fareed.ahmad@uvas.edu.pk for data relating to this study.

## Abbreviations

BO: Bayesian optimization
Ca: Calcium
Cd: Cadmium
Chi-Sq: Chi-square
Co: Cobalt
Cr: Chromium
Cu: Copper
cy: Clay
EM: Ensemble model
Fe: Iron
Ft: *Francisella tularensis*
GI: Gini-Index
GP: Gaussian process
K: Potassium
Mg: Magnesium
Mn: Manganese
MO: Moisture
N: Nitrogen
Na: Sodium
Ni: Nickel
NN: Neural networks
OM: Organic matter
P: Phosphorus
Pb: Lead
RLF: ReliefF
RS: Random search
Si: Silt
SS: Soluble salt
SVM: Support vector machine
