# Supplementary figures and images for: Identifying key soil characteristics for Francisella tularensis classification with optimized Machine learning models
Source: Sci Rep. 2024 Jan 19;14:1743. doi: 10.1038/s41598-024-51502-z (PMC10799052; doi:10.1038/s41598-024-51502-z)

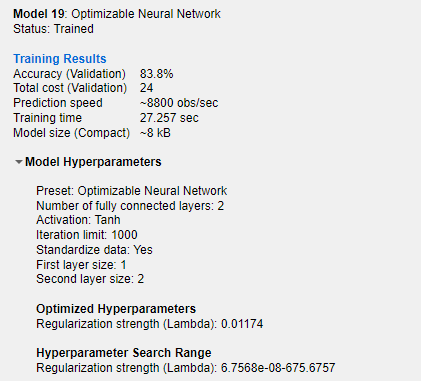


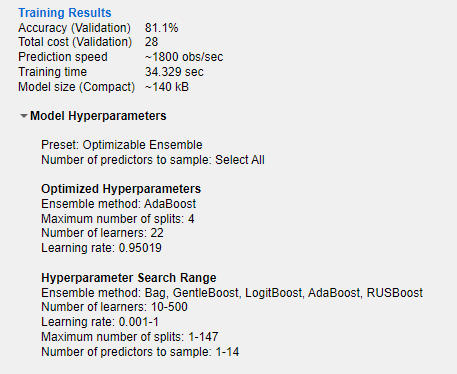

Supplement: Supplementary file 2 — Supplementary Information 2. [file 41598_2024_51502_MOESM2_ESM.docx]
